# TRC120038, a Novel Dual AT_**1**_/ET_**A**_ Receptor Blocker for Control of Hypertension, Diabetic Nephropathy, and Cardiomyopathy in ob-ZSF1 Rats

**DOI:** 10.4061/2011/751513

**Published:** 2011-12-22

**Authors:** Anookh Mohanan, Ram Gupta, Amita Dubey, Vikrant Jagtap, Appaji Mandhare, Ramesh C. Gupta, Vijay Chauthaiwale, Chaitanya Dutt

**Affiliations:** Torrent Research Centre, Torrent Pharmaceuticals Ltd., Gujarat, Gandhinagar 382428, India

## Abstract

In hypertensive subjects, angiotensin II and endothelin participate in a manner involving closely interwoven pathways in increasing blood pressure (BP) and inducing end organ damage. The primary objective of this study was to determine the effect of TRC120038, a novel dual AT_1_/ET_A_ receptor blocker on BP, in obese Zucker spontaneously hypertensive fatty rats (ob-ZSF1), an animal model of moderate hypertension, diabetes with progressive renal and cardiac dysfunction. Ob-ZSF1 rats loaded with 0.5% salt were treated with TRC120038 (11.8 mg/kg bid.) or candesartan cilexetil (0.3 mg/kg od.) or vehicle control. Blood pressure (by radio-telemetry) and renal functional markers were monitored throughout the study. Cardiac function was assessed terminally by pressure volume catheter. Markers for renal dysfunction were measured and changes were evaluated histopathologically. TRC120038 showed greater fall in both systolic and diastolic BP in comparison to candesartan at its maximum antihypertensive dose. TRC120038 also reduced the severity of renal dysfunction and preserved cardiac function in ob-ZSF1 rat.

## 1. Introduction

Inspite of multihypertensive drug treatment, significant proportion of poorly controlled hypertensives exist [1–3]. Greater incidences of stroke, heart failure, and end stage renal disease are reported in these patients [4, 5]; rigorous blood pressure (BP) lowering treatment targets are recommended for these patient population [6, 7].

Hypertensive patients have upregulated endothelin and renin angiotensin system (RAS). This has been clearly shown by elevated plasma levels of endothelin-1 (ET-1) [8] and plasma renin activity [9] in such subjects. More recently, endothelin receptor blockers have shown to be effective in resistant hypertensives [10]. It is now well established that increased ET-1 activity is one of the contributors for increased incidence of hypertension in diabetics with insulin resistance [11–13]. Similarly, association of increased body mass index with enhanced-ET_A_-receptor-dependent vasoconstrictor activity in hypertensive subjects has been demonstrated [13–15]. A substantial part of the poor responders to current antihypertensive therapy is characterized by salt sensitivity and increased-ET_A_-receptor-dependent vasoconstrictor tone [16, 17]. Such evidence suggests that abnormality in the endothelin system (in addition to RAS) plays a role in the pathophysiology of poorly controlled/difficult to treat (obesity- and diabetic-related) hypertension and that targeting endothelin system in addition to the RAS is a useful treatment for hypertension in these patients.

Diabetic nephropathy, characterized by progressive rise in BP, persistent albuminuria, and declining glomerular filtration rate, remains the leading cause of end stage renal disease requiring renal replacement therapy. Clinical evidence suggests that there is a large unmet need to develop strategies for prevention of diabetic nephropathy and its progression to end stage renal disease [18, 19].

Role of RAS in development of glomerular hypertension and nephropathy is well established [20, 21]. Targeting RAS through angiotensin receptor blockade is now a strategy of choice to treat hypertension. This strategy has also shown some promises in diabetic nephropathy. In addition, ET-1 stimulates hypertrophy, proliferation, and extracellular matrix accumulation in the kidney [22–24], which are prevented by ET_A_ receptors blockers [25, 26]. Similarly, ET_A_ receptor blockade has shown antiproteinuric effects in proteinuric chronic kidney disease patients [27].

Emerging experimental evidence from various studies suggests that angiotensin II (Ang II) and endothelin participate in a manner involving closely interwoven pathways (crosstalk) in increasing BP and inducing end organ damage [28–35]. The pharmacological benefits of dual Ang II and ET-1 blockade have been demonstrated in form of their anti-hypertensive and anti-proteinuric effects both in rodents and humans [26, 36–39]. Hence, concomitant blockade of both angiotensin and endothelin endocrine/paracrine pathways may lead not only to enhanced BP reductions but also retard end organ damage directly or indirectly. Molecules with dual activity (dual receptor blockers) are like fixed-dose combination of two drugs which generally has the limitation of inability for physicians to titrate the individual dose of the combination for differential individual activity. At the same time, adopting dual receptor blockers strategy offers unique advantages especially when both the actives are contributing to overall similar therapeutic effect. Better patient compliance and lower production cost further add utility of such approach in appropriate scenarios.

These studies thus provided a rationale for a therapeutic strategy to develop compounds with dual receptor blocker action against AT_1_ and ET_A_ receptors. Torrent's discovery program identified TRC120038, a novel dual AT_1_/ET_A_ receptor blocker. Candesartan cilexetil (candesartan), a potent, long-acting, unsurmountable AT_1_ receptor blocker used in clinic, with once-a-day dosing was selected to be studied as the comparator to TRC120038. This study was thus performed to compare the therapeutic potential of TRC120038 and candesartan in reducing BP and end organ damage (diabetic nephropathy and cardiomyopathy) in obese Zucker spontaneously hypertensive fatty rats (ob-ZSF1), an animal model with a severe form of diabetes associated with clinically relevant comorbidities eventually leading to progressive renal and cardiac dysfunction [40].

## 2. Methods

### 2.1. Test Compounds

**Figure 1:**
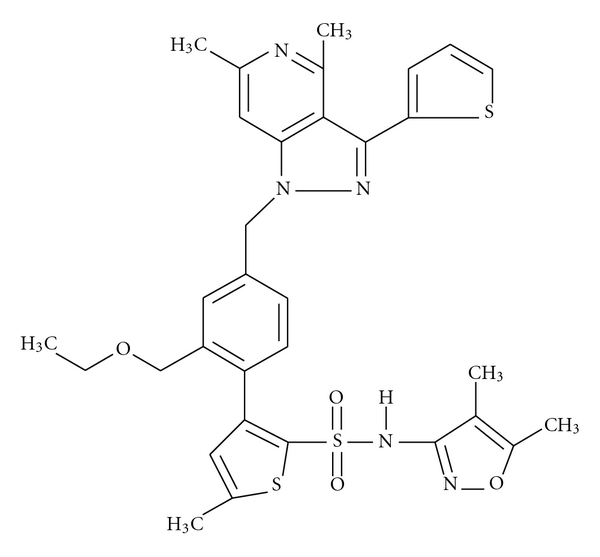
Chemical structure of TRC120038.

The chemical name of TRC120038 is *N*-(4,5-dimethyl-1,2-oxazol-3-yl)-3-[4-{[4,6-dimethyl-3-(thiophen-2-yl)-1*H*-pyrazolo[4,3-*c*]pyridin-1-yl]methyl}-2-(ethoxymethyl) phenyl]-5-methylthiophene-2-sulfonamide, and its chemical structure is presented in Figure 1. Patent was filed and published under PCT (WO 2007/100295 A1). This compound was synthesized by Torrent Pharmaceuticals Ltd. TRC120038 and candesartan were formulated as suspensions in 3% w/v hydroxyl propyl cellulose in water. TRC120038, a novel dual AT_1_/ET_A_ receptor blocker has an *in vitro* EC_50_ of 3 nM and 158 nM for hAT_1_ and hET_A_ receptor blockade, respectively, along with reasonable selectivity (>50x) against hAT_2_ and hET_B_ receptors. TRC120038 has no significant binding to any other receptors nor does it alter the activity of any enzymes out of a wide battery of receptors and enzymes tested on the PanLabs battery of assays.

### 2.2. Animals

Male ob-ZSF1 rats (Charles River Laboratories, USA) were used in the study. All animals were maintained in the Pre-clinical and Safety Evaluation Department, Torrent Research Centre, housed in individually ventilated cages (IVC) system, maintained on a 12-hour light-dark cycle, and had access to Purina 5008 rat chow and water *ad libitum*. The IVCs were maintained under controlled environment temperature (22 ± 3°C), relative humidity (30–70%), and air exchange rate (40–50 air changes per hour). All animal experiments were conducted in accordance with the CPCSEA guidelines, and the studies were approved by the Institutional Animal Ethics Committee.

### 2.3. Hemodynamic Measurements

Rats (180–250 gm b.wt; 6–8 weeks of age) were implanted with radio-telemetry transmitters (TA11PA-C40, Data Sciences, St. Paul, Minn, USA) under 1.5% isoflurane anesthesia, and the abdominal aorta was cannulated to place the tip of the gel-filled transmitter catheter, caudal to the renal arteries for arterial BP recording. BP data were collected with a computer-driven data acquisition system (Dataquest A.R.T. 3.01, Data Sciences); the acquisition software was appropriately configured to record parameters for 10 seconds every 10 minutes, continuously for 21 hours. The mean of 0–19-hour data of BP recording of each day was calculated as the average BP for the day. The BP of the rats was recorded once weekly for 21 hours from 10 weeks of age till study termination at around 41 weeks of age.

Rats at around 10 weeks of age were given 0.25% sodium chloride (salt) dissolved in drinking water for a week and then salt load increased to 0.5% (85.5 mmol/L) for the rest of the study, to accelerate the vasculopathy and cardiomyopathy.

### 2.4. Antihypertensive Activity of TRC120038 and Candesartan Dose Response

To establish the optimal treatment dose of TRC120038 and candesartan for the chronic efficacy study, rats at around 12 weeks of age were randomized into six treatment groups (*n* = 6 in each group), with matching baseline mean blood pressure (MBP). On the first day of treatment, all rats in the six treatment groups were administered vehicle (1 mL/kg) by oral route; on the subsequent two days, three treatment groups were administered TRC120038 (1.26, 11.8, 45.5 mg/kg p.o., bid.) and the other three groups received candesartan (0.03, 0.3 and 1 mg/kg p.o., od.) in the morning and vehicle 12 hours later in the evening. These doses of TRC120038 and candesartan were derived from efficacy studies done earlier in our laboratory.

### 2.5. Repeat Dose Efficacy Study with TRC120038 and Candesartan

#### 2.5.1. Drug Treatment

At the age of around 15 weeks, the rats were randomly distributed into three treatment groups; (*n* = 8–11 per group) on the basis of baseline MBP. Group1: control group was administered vehicle (1 mL/kg p.o., bid.); group 2: TRC120038 group was administered 11.8 mg/kg, p.o., bid. and group 3: candesartan group was administered 0.3 mg/kg, p.o., od. until study termination at around 41 weeks of age (25 weeks of treatment).

#### 2.5.2. Estimation of Various Urine Biochemical Parameters

The urine albumin, creatinine, and total protein were monitored, before treatment initiation (treatment month 0, i.e., 15 week of age) and were repeated monthly thereafter. Urine samples were collected every 2 hours and stored at 2–4°C before pooling 24-hour samples. Total protein and creatinine were estimated using fully automated clinical chemistry analyzer Olympus AU400 (Olympus Corporation, Japan). Urinary albumin estimation was performed in accordance with in-house method developed for estimating albumin content in urine sample by HPLC.

#### 2.5.3. Measurement of Left Ventricular (Cardiac) Performance

Terminal measurement of left ventricular (LV) function was performed using Millar Pressure Volume (PV) System (Millar Instruments, Houston, Tex, USA). A microtip PV catheter (SPR-838) was inserted into the right carotid artery and advanced into the LV. Polyethylene catheter was inserted into the left jugular for fluid administration. After stabilization for 10–20 minutes, LV-PV signals were recorded continuously at a sampling rate of 1000/second using an MPVS-300 conductance system (Millar Instruments) coupled to a PowerLab 8/30 (ADInstruments, Australia). 50 *μ*L of 20% saline was injected intravenously so as to establish parallel conductance volume from shift of PV relations, and this was used for correction of the cardiac mass volume. LV-PV relations were also captured by transiently compressing the inferior vena cava.

LV parameters were computed using cardiac PV analysis software (PVAN3.2, Millar Instruments) as described previously [41, 42]. Volume calibrations were performed with Millar volume calibration cuvette which consists of a 1 cm deep cylindrical block with cylindrical holes of known diameters ranging from 2 to 11 mm filled with fresh heparinised whole rat blood. The linear volume-conductance regression of the absolute volume in each cylinder versus the raw signal acquired by the conductance catheter was used for the volume calibration using PVAN 3.2. Cardiac output was normalized to body weight [cardiac index (CI)]. The total peripheral resistance index (TPRI) was calculated by the following equation: TPRI = MBP/CI [43]. Augmentation index (AI) was calculated by method suggested by Westerhof et al. [44].

After measurement of cardiac performance, the rats were sacrificed for detailed necropsy and the kidneys were collected and stored for histopathological examination.

#### 2.5.4. Histopathological Examination

Kidneys collected were subjected to gross and microscopic histopathological examination. Tissues were fixed in 10% neutral buffer formalin. Approximately, 2-3 mm thick kidney transverse and longitudinal sections were dehydrated in graded isopropyl alcohol, cleared in xylene, and infiltrated in paraffin [45]. Three serial sections of tissue (4 *μ*m) embedded in paraffin were taken on the glass slides. Sections were stained with routine hematoxylin and eosin stain, with Masson's trichrome stain for connective tissue, and with periodic acid Schiff stain (PAS) [45] to study glomerulosclerosis. Kidney pathology was evaluated under subheading of glomerulosclerotic index (GSI), tubulopathy, interstitial fibrosis, and media to lumen ratio in intra renal artery [40, 46, 47]. For GSI, 100 glomeruli from each animal were scored from one end to another; care was taken to avoid the duplication of glomeruli.

All the microscopic changes were evaluated in blinded fashion in grade scale of 0–4 under the light microscopy (Leica DM2500) [48], where grade 0 defines no change in tissue, grade 1-minimal (11–25%); grade 2-mild (26–50%); grade 3 moderate (51–75%) and grade 4 severe (>75%) pathological changes in the tissues.

For thickness of basement membrane of parietal layer of Bowman's capsule: 25 glomeruli (complete midline exposure) were photographed under 400x (Leica DM2500) from each kidney, and parietal layer of Bowman's capsule width (*μ*m) was measured at 4 random positions in each glomerulus by calibrated LAS software. Cross-section of intrarenal artery (arcurate and intralobular) at corticomedullary junction of transverse section of kidney (near-to-middle area) was photographed using Leica DM2500 microscope with attached CCD camera DFC295 (~30–40% rats in each group). Care was taken to select the near-to-circular cross-section of artery. Cross-sectional area of arterial lumen and medial wall thickness was measured by image analysis software (Image-pro Plus Version 6.0). Ratio of media-to-lumen area was calculated [47].

#### 2.5.5. Pharmacokinetic Monitoring

Oral pharmacokinetic (PK) profiling of TRC120038 and candesartan was performed in a satellite group of nontelemetry transmitter implanted male ob-ZSF1 rats (*n* = 4 in each group), being treated similarly to those implanted with telemetry transmitters. PK profiling was performed on day 1 of treatment and again at end of 4 months (18 weeks) of treatment. On the day of PK study, the second evening dose of TRC120038 was not administered to capture the 24-hours PK profile.

### 2.6. Statistical Analysis

Results are expressed as mean ± SEM for MBP. Statistical comparisons between and within groups were conducted using analysis of variance by repeated measures (RMANOVA). One-way ANOVA with multiple comparisons using Tukey as post hoc test was used for comparisons amongst groups. Graded data of kidney histopathological analysis was subjected to nonparametric chi-square test. Values exceeding 95% critical limits (*P* ≤ 0.05) are considered to be statistically significant. Statistical analysis has been performed using statistical analysis system (SAS, Version-9.1) and GraphPad Prism (version 3.0).

## 3. Results

### 3.1. Antihypertensive Activity of TRC120038 and Candesartan Dose Response

**Figure 2:**
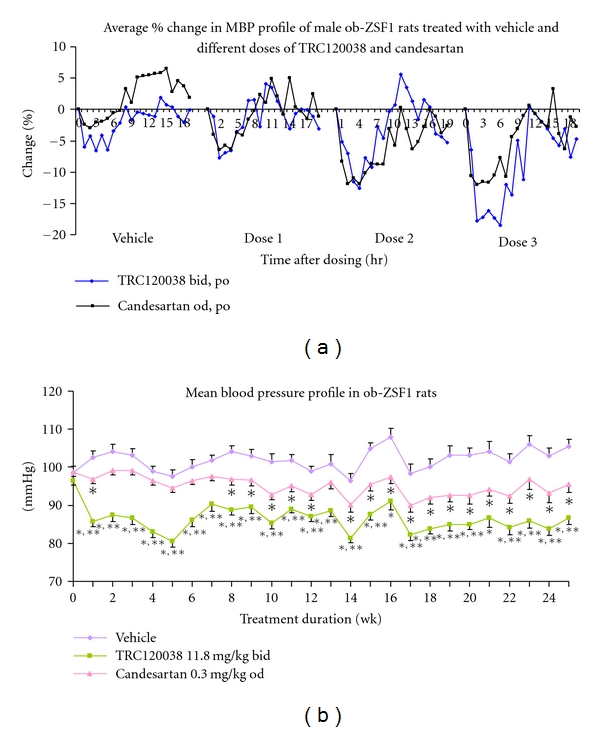
(a) Percent change in MBP over 19 hr after dosing at 12 weeks of age in male ob-ZSF1 rats treated with vehicle and different doses of TRC120038 (dose 1: 1.26 mg/kg; dose 2: 11.8 mg/kg; dose 3: 45.5 mg/kg, p.o., bid.) and candesartan (dose 1: 0.03 mg/kg; dose 2: 0.3 mg/kg; dose 3: 1.0 mg/kg, p.o., od.). *n* = 6 in each treatment group. *P* ≤ 0.05 vehicle versus candesartan dose 2 and 3 and vehicle versus TRC120038 dose 3 by RMANOVA. (b) Weekly average MBP profile (mean of 0–19 hr) in male ob-ZSF1 rats upon treatment with vehicle, TRC120038, or candesartan (*n* = 8, 11, and 10 resp.) for 25 weeks. *P ≤ 0.05 versus vehicle; **P ≤ 0.05 TRC120038 versus candesartan by one way ANOVA with multiple comparison using Tukey post hoc test. Also, *P* ≤ 0.05 for vehicle versus TRC120038 treatment and TRC120038 versus candesartan treatment by RMANOVA.

A dose-dependent fall in MBP was observed with three incremental doses of TRC120038, whereas maximum fall in MBP with candesartan was observed with its second dose (0.3 mg/kg) itself (Figure 2(a)). Dose response revealed that AUC_(0–19 hr)_ for net MBP change (691.5 ± 40.9 mmHg·hr) seen with dose 2 of TRC120038 (11.8 mg/kg) was similar to that observed with maximal effective dose 2 of candesartan (0.3 mg/kg) (662.8 ± 13.1 mmHg·hr).

### 3.2. Repeat Dose Efficacy Study with TRC120038 and Candesartan

#### 3.2.1. Antihypertensive Activity of TRC120038 and Candesartan

Ob-ZSF1 rats followed up for 25 weeks of treatment and maintained on 0.5% salt in drinking water displayed a modest increase in MBP. At 25 weeks of treatment with vehicle, a rise of 16.4 mmHg and 3 mmHg in systolic blood pressure (SBP) and diastolic blood pressure (DBP), respectively, was observed. The rise in MBP was prevented by candesartan, whereas TRC120038 was successful in further lowering the MBP across time in comparison to levels before initiation of treatment (Figure 2(b)). TRC120038 within seven days of treatment produced significantly greater fall in MBP in comparison to vehicle and candesartan (Figure 2(b)), and this trend continued throughout the study duration, of 25 weeks. Though both TRC120038 and candesartan were able to prevent rise and maintain lower SBP and DBP, the magnitude of fall in SBP and DBP brought about by TRC120038 treatment was greater in comparison to candesartan treatment (23.6 versus 16.1 mmHg for SBP and 13.1 versus 7.5 mmHg for DBP, resp.).

#### 3.2.2. Effect on Left Ventricular Performance

**Figure 3:**
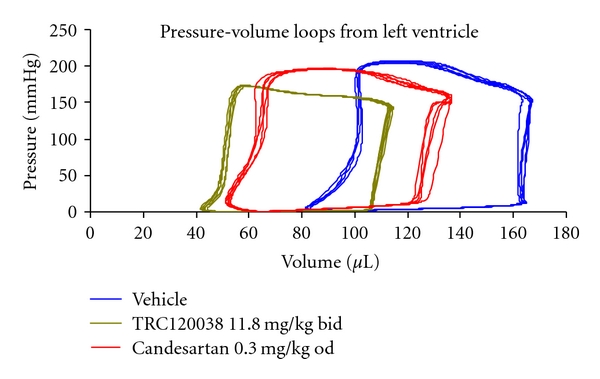
Representative pressure-volume loops from left ventricle of male ob-ZSF1 rats treated with vehicle, TRC120038, or candesartan.

Representative LV-PV loops and effect of treatment on LV functional parameters are shown in Figure 3 and Table 1, respectively. Evaluation of cardiac function revealed ability of TRC120038 to significantly prevent rise in indices of preload (end diastolic pressure (EDP) and end diastolic volume (EDV)) and afterload (end systolic volume (ESV), arterial elastance, and TPRI); prevent deterioration in diastolic function as evident from better preserved time constant of LV pressure decay (tau), lesser EDP; additionally, the minimum pressure during cardiac cycle in diastole was significantly less in comparison to vehicle-treated group. Further, the reduced AI in TRC120038-treatment group indicates ability of TRC120038 to prevent rise in arterial stiffness in comparison to vehicle treated ob-ZSF1 rats. Also, TRC120038 treated group was found significantly better to candesartan treated group in preventing rise in preload (EDV), cardiac performance [ejection fraction (EF)] and a trend towards better preserved afterload [end systolic pressure (ESP)] and AI.

#### 3.2.3. Renal Effect

**Figure 4:**
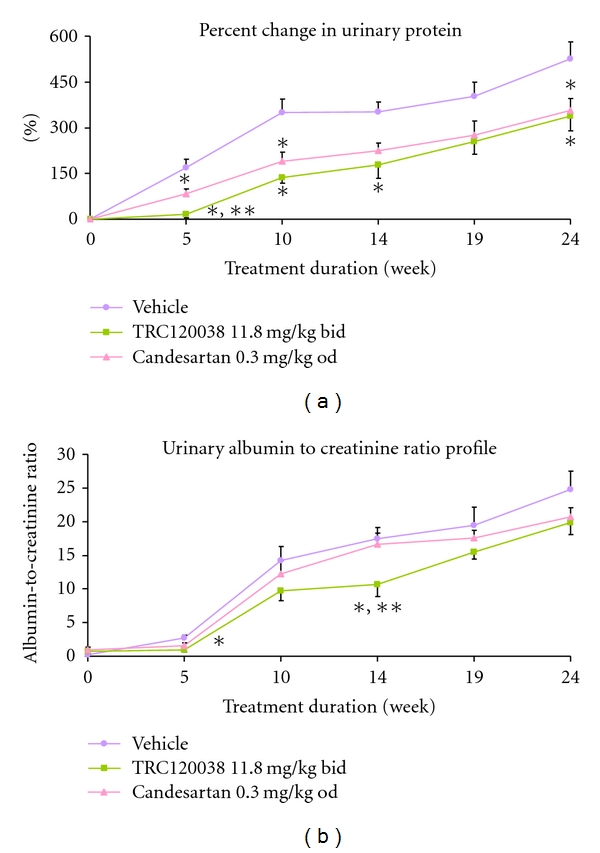
(a) Percent change from basal levels in urinary protein excretion profile of male ob-ZSF1 rats treated with vehicle, TRC120038, or candesartan. *P ≤ 0.05 versus vehicle; **P ≤ 0.05 TRC120038 versus candesartan by one-way ANOVA with multiple comparison using Tukey post hoc test. (b) Urinary albumin to creatinine ratio profile of male ob-ZSF1 rats treated with vehicle, TRC120038, or candesartan. *P ≤ 0.05 versus vehicle; **P ≤ 0.05 TRC120038 versus candesartan by one-way ANOVA with multiple comparison using Tukey post hoc test.

Percent increases from baseline (before treatment initiation) for urinary protein and rise in albumin to creatinine ratio, over the treatment duration, were significantly prevented in the TRC120038 treated group as compared to vehicle control as shown in Figures 4(a) and 4(b). Though similar functional improvement in percent change in urinary protein excretion was observed with candesartan treatment a better trend was still evident with TRC120038 treatment reaching significance at some points (Figure 4(a)).

#### 3.2.4. Histopathological Changes in Kidney

Histopathological examination of kidney from ob-ZSF1 rats revealed signs of moderate-degree nephropathy. Focal-to-global sclerosis of glomeruli, characterized by expansion of mesangial matrix, thickened basement membrane, hypercellularity, hypertrophied podocytes with/without pseudo cyst formation, foam cells infiltration, and obliteration of Bowman's space resulting in sclerosed or obsolesce glomeruli were observed in all the rats. Tubulopathy included dilated tubules with loss of brush border, desquamation of tubular epithelium, hyperplastic/hypertrophic tubular epithelium or flattened epithelial lining and hyaline cast in tubular lumen. In more severe cases, atrophic and degenerating tubules along with regenerating tubules were also observed. Interstitium showed increase in the interstitial space, fibrous tissue proliferation, and infiltration of mono nuclear cells.

**Figure 5:**
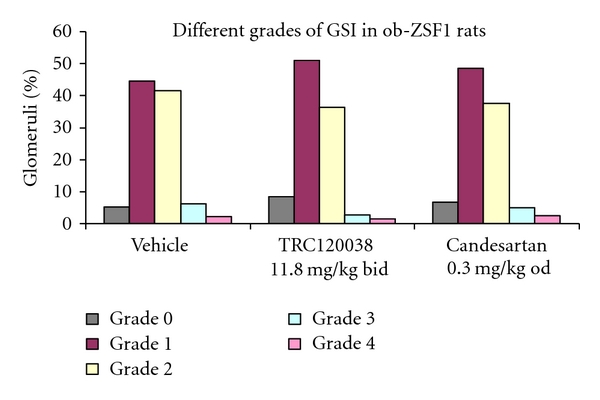
Percent incidence of glomeruli in different grades of GSI for ob-ZSF1 rats treated with vehicle, TRC120038, or candesartan. Incidences are significantly different (**P** ≤ 0.01) for TRC120038 versus candesartan and vehicle versus TRC120038 by chi-square test.

**Figure 6:**
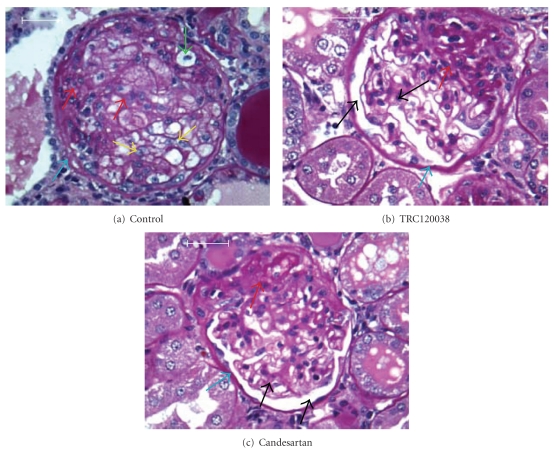
(a) Glomerulus from vehicle-treated ob-ZSF1 rat, showing global glomerulo sclerosis, expansion of mesangial matrix (red arrow), infiltration of foamy macrophage (green arrow), podocyte hypertrophy (yellow arrow), thickening of basement membrane (blue arrow), and complete obliteration of Bowman's space. PAS staining, 400x (b) Glomerulus from TRC120038-treated ob-ZSF1 rat. (c) Glomerulus from candesartan treated ob-ZSF1 rat, showing normal glomerulus portion with Bowman's space (black arrow), focal glomerulosclerosis, focal expansion of mesangial matrix with hypercellularity (red arrow), and reduced thickening of basement membrane (blue arrow). PAS staining, 400x.

**Figure 7:**
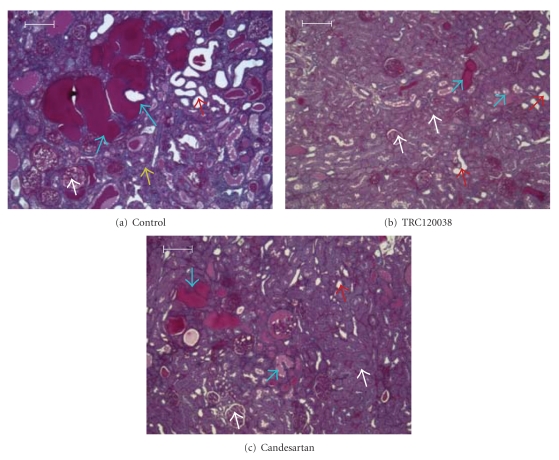
(a) Kidney from vehicle-treated ob-ZSF1 rat, showing more number of dilated tubules (red arrow), tubules with hyaline cast (blue arrow), sclerosed glomeruli (white arrow), and interstitial inflammation (yellow arrow). PAS staining, 40x (b) Kidney from TRC120038. (c) Candesartan-treated ob-ZSF1 rat, showing normal tubules and glomeruli (white arrow), reduced number of dilated tubules (red arrow), and tubules with cast (blue arrow). PAS staining, 40x.

Treatment with TRC120038 significantly prevented the deterioration in GSI as evident from greater percentages of normal glomeruli and glomeruli with minimal sclerotic changes in comparison to glomeruli of vehicle and candesartan-treated rats (Figures 5 and 6). Similarly, both the treatment groups (TRC120038: 4.07 ± 0.49 *μ*m and candesartan: 4.06 ± 0.98 *μ*m) significantly prevented increase in the width of parietal layer of Bowman's capsule as compared to vehicle-treated rats (5.08 ± 1.31 *μ*m). Further, a clear trend indicating less severe grade of tubules with cast and better maintained normal structure of tubules were observed in TRC120038- (1.65 ± 0.15 and 2.04 ± 0.18, resp.) and candesartan- (1.79 ± 0.12 and 2.21 ± 0.16, resp.) treated rats as compared to the vehicle-treated (2.18 ± 0.26 and 1.91 ± 0.25, resp.) ob-ZSF1 rats (Figure 7). Masson trichome stained kidney section showed the presence of interstitial fibrosis in all ob-ZSF1 rats. TRC120038-treated ob-ZSF1 rats had lesser severity (1.23 ± 0.12) of renal interstitial fibrosis in comparison to the vehicle-treated rats (1.55 ± 0.17) and candesartan-treated rats (1.50 ± 0.19).

Though not significant, a positive trend towards TRC120038 treatment-related improvement was observed in the media-to-lumen ratio of intrarenal artery with respect to other two treatment groups.

#### 3.2.5. Pharmacokinetic Measurements

 AUC_0–*α*_ was 13189 ± 1859 and 9266 ± 1028 hr·ng/mL, with *C *_max_ 2610 ± 275 and 2500 ± 858 ng/mL on day 1 and after 4-month repeated administration, respectively, showing no accumulation of TRC120038 on repeated dosing. Similarly, exposure to candesartan, determined on first day of treatment and determined again after multiple dosing for 4 months, was similar with AUC_0–*α*_ 386.3 ± 40.9, 335.2 ± 9.4 hr·ng/mL and *C *_max_ 55.3 ± 5 and 56.8 ± 8.4 ng/mL, respectively.

## 4. Discussion

In this study, TRC120038, a novel dual AT_1_/ET_A_ receptor blocker, on repeated administration, has clearly prevented increase in BP, attenuated progression in the severity of renal dysfunction, and preserved cardiac function in ob-ZSF1, an animal model of metabolic syndrome.

In present study, the anti-hypertensive and cardiorenal protective effects of our dual AT_1_/ET_A_ receptor blocker TRC120038 and a well-established angiotensin receptor blocker candesartan used clinically as an anti-hypertensive at a dose which produces almost similar fall in BP were studied. Dose response generated upon two days of treatment with TRC120038 or candesartan revealed that candesartan produced its maximal anti-hypertensive effect in salt-loaded ob-ZSF1 rats at a dose of 0.3 mg/kg, p.o., od., and TRC120038 produced almost similar fall in BP at a dose of 11.8 mg/kg, p.o., bid. These doses were thus selected for subsequent comparison of efficacy (BP and cardiorenal damage) upon chronic administration of TRC120038 and candesartan in ob-ZSF1 rats. Decision to administer TRC120038 twice a day was based on its PK profile. The dose-response study shows greater fall in BP with higher doses of TRC120038 as compared to candesartan. At the studied doses, saturation in candesartan's efficacy is seen. The greater fall in BP is probably due to the additional contribution from the ET_A_ receptor blockade component of TRC120038.

Here, TRC120038 upon 25 weeks chronic treatment clearly proved to be a better anti-hypertensive and in addition, preserved cardiac function and reduced the severity of renal dysfunction in ob-ZSF1 rats. This additional BP lowering seen on chronic treatment by TRC120038 in comparison to candesartan could be related to its ET_A_-receptor-blocking component which addresses the activated endothelin system in salt loaded ob-ZSF1 rats. This is also in line with the observations that diabetic and obese patients have higher ET-1 activity [12, 14]. The possibility of additional improvement observed with TRC120038 due to its probable increased systemic exposure with time was ruled out by the observation where exposure to TRC120038 and candesartan, determined on first day of treatment, was similar to that observed at the end of 18 weeks of repeated dosing.

Diabetic nephropathy is likely to manifest with comorbidities like hypertension, cardiomyopathy, and other microvascular complications, and these patients would be on multiple drug therapy which is high likely to include an angiotensin-converting enzyme (ACE) inhibitor/AT_1_ receptor blocker. Though used as a first-line therapy, intervening RAS at the level of ACE or AT_1_ receptors in renal patients is shown not to consistently reduce proteinuria, which is not only a powerful predictor but also a promoter of renal dysfunction; this is true for the dosages recommended for hypertension control and even for supramaximal dosages [49].

High levels of endothelin are reported in diabetic nephropathy [50, 51] and diabetic cardiomyopathies [52–54] and also showed the beneficial effect of endothelin receptor blocker in cardio-renal syndrome [55]. Similarly, as expected theoretically, combination of selective ET_A_ receptor blocker and ACE inhibitor has produced impressive benefit, including regression of lesions in model of diabetic nephropathy [56]. These findings fully justify the notion that endothelin receptor blocker holds much promise for the management of diabetic nephropathy.

Dual inhibition of RAS and endothelin system holds promise in diabetic nephropathy as pharmacologic blockade of RAS and endothelin independently has been shown to ameliorate the abnormality in glomerular permeability selectivity [57–59]. Studies have shown BP-independent effect of pharmacological blockade of RAS and endothelin system in reducing indices of renal injury as compared to the respective monotherapies [27, 60, 61]. In addition, protein loading of proximal tubular cells is suggested to provoke tubular expression of ET-1 and secretion of ET-1 [62, 63]. This hypothesis is in good agreement with the finding of studies on the expression of ET-1 and endothelin receptors in human renal biopsy specimens, which demonstrated more pronounced expression in individuals with marked proteinuria [64].

Ob-ZSF1 rats display all characteristics of diabetic nephropathy, which becomes even more severe as model progresses in age and ultimately animals are reported to die of nephropathy by age of around one year [40, 46, 65]. Similar were our observations where severity of diabetic nephropathy was clearly evident by the extent and rate of increase of proteinuria and terminal histopathological examination of kidney.

At the start of study, overt hyperglycemia and proteinurea were seen in these animals (12 wks of age) though hypertension was mild, which leads to the fact that hypertension has minimal role in development of nephropathy in this model. In our study, treatment with TRC120038 resulted into better reduction in urinary-albumin-to-creatinine ratio and protein excretion in comparison to candesartan-treated ob-ZSF1 rats. These functional benefits are expected to translate into histopathological findings as well, since excretion of proteins has been suggested to have toxic effect on tubular epithelial cells and may be responsible for the initiation of interstitial inflammation and scarring [66, 67]. This correlates well in our case as TRC120038 treatment was found superior to even candesartan in having less severe GSI and tubulo-interstitial pathology. Thus, in our study, functional improvement in the ability of kidney to prevent excessive urinary protein excretion with treatment is well correlated with histopathological findings of better preserved microscopic structures of kidney.

Major clinical trials with ACE inhibitors have shown fewer cardiovascular events and a lower rate of macrovascular complications in high-risk diabetic patients [68], suggesting cardioprotective potential of intervening RAS in such patient population. Evidence is also accumulating which supports possible importance of the overactive endothelin system in the pathogenesis of diabetic cardiovascular complications. In hearts from experimental animals with chemically induced diabetes, mRNA and protein for ET-1 and endothelin receptors were elevated [68]. The increased expression of mRNA encoding for ET-1 and endothelin receptors was associated with myocardial cell death, focal scarring of the myocardium, and increased expression of several extracellular matrix proteins. All these insults were mitigated by an endothelin receptor blocker [69]. Similarly, in diabetic animals, endothelin receptor blockade limited the rise in BP [70] and antagonized myocardial contractile depression [71], macrovascular endothelial dysfunction, and renal target organ damage [72].

The hallmark of diabetic cardiomyopathy which includes diastolic and systolic dysfunctions is also related to change in cardiac preload and afterload. TRC120038 successfully retarded rise in preload and afterload parameters and also prevented rise in peripheral resistance and stiffness of conduit arteries which is reflected well in parameters as TPRI and AI, respectively. Though not always significant, TRC120038 proved superior to candesartan in preservation of the LV parameters studied. However, no differences were observed in any of the LV parameters captured after transient occlusion of inferior vena cava which reflects preload-independent functioning of LV. It is possible that by the end of treatment duration of 25 weeks significant structural changes in myocardium were yet to set in. This is also supported by no significant difference observed in the heart weight and other morphometric analysis which includes length and width of heart.

A direct correlation between high BP and end organ damage (heart, kidney, and brain) has been long established and also proven is the fact that lowering BP directly correlates with lesser damage to end organs [73]. The better cardio-renal protective effects seen with TRC120038 have significant contributions from their superior BP control compared to candesartan, and an additional contribution from blockade of tissue ET in addition to tissue RAS cannot be ruled out.

## 5. Conclusion

In conclusion, TRC120038, a dual AT_1_/ET_A_ receptor blocker, has clearly shown its multifaceted potential in controlling hypertension and attenuating diabetic end organ damage in a way similar, or even better (in several aspects) than candesartan. Evidence, like these, provides strong background for the exploration for dual RAS and endothelin system blockade for treatment of poorly controlled hypertension and diabetic end organ damage. Our study with novel dual AT_1_/ET_A_ receptor blocker TRC120038 provides experimental evidence for the benefits of such an approach. However, outcome of further detailed safety and toxicity studies would be required to shape the future exploration of this molecule in target human population.

## Figures and Tables

**Table 1:** Terminal left ventricular functional parameters of male ob-ZSF1 rats treated with vehicle, TRC120038, or candesartan.

Left Ventricular functional Parameters	Vehicle 1 mL/kg	TRC120038 11.8 mg/kg, bid, po	Candesartan 0.3 mg/kg, od, po
	Mean ± SEM (*n* = 8)	Mean ± SEM (*n* = 11)	Mean ± SEM (*n* = 10)
Heart rate (bpm)	264.1 ± 9.1	274.1 ± 14.1	266.4 ± 17.1
Minimum volume (*μ*L)	80.8 ± 14.7	38.0 ± 8.4^∗,∗∗^	82.0 ± 11.8
End systolic volume (*μ*L)	97.2 ± 14.1	48.0 ± 11.1^∗,∗∗^	91.9 ± 10.8
End diastolic volume (*μ*L)	139.7 ± 19.5	101.0 ± 12.8^∗,∗∗^	144.0 ± 13.5
End systolic pressure (mmHg)	180.4 ± 10.3	133.0 ± 11.9*	149.2 ± 11.7*
End diastolic pressure (mmHg)	13.7 ± 3.1	7.5 ± 1.0*	7.2 ± 0.8*
Stroke volume (*μ*L)	65.4 ± 7.2	73.0 ± 8.7	72.4 ± 4.2
Ejection fraction (%)	46.7 ± 4.8	69.4 ± 5.0^∗,∗∗^	48.7 ± 3.6
Cardiac output (*μ*L/min)	17143.6 ± 1815.0	20298.4 ± 2920.0	19018.4 ± 1241.0
Arterial elastance (mmHg/*μ*L)	3.0 ± 0.4	2.1 ± 0.3*	2.1 ± 0.2*
Tau_w (msec)	15.9 ± 1.0	12.2 ± 0.5*	12.8 ± 0.6*
Total peripheral resistance index	5.4 ± 0.8	3.6 ± 0.6*	3.9 ± 0.4*
Augmentation Index	0.4 ± 0.04	0.3 ± 0.04*	0.4 ± 0.05

*P ≤ 0.05 versus vehicle; **P ≤ 0.05 versus candesartan by Student's *t*-test.

Data represent mean ± SEM (standard error mean).

## References

[1] Chobanian AV, Bakris GL, Black HR (2003). The seventh report of the joint national committee on prevention, detection, evaluation, and treatment of high blood pressure: the JNC 7 report. *Journal of the American Medical Association*.

[2] Roger VL, Go AS, Lloyd-Jones DM (2011). Heart disease and stroke statistics—2011 update: a report from the American Heart Association. *Circulation*.

[3] Persell SD (2011). Prevalence of resistant hypertension in the United States, 2003–2008. *Hypertension*.

[4] Epstein BJ (2008). Efficacy and safety of darusentan: a novel endothelin receptor antagonist. *Annals of Pharmacotherapy*.

[5] Rodgers A, MacMahon S, Yee T, Clark T (1998). Blood pressure, cholesterol, and stroke in eastern Asia. *Lancet*.

[6] Arauz-Pacheo C, Parrott MA, Raskin P (2003). Treatment of hypertension in adults with diabetes. *Diabetes Care*.

[7] National Kidney Foundation Guideline (2002). K/DOQI clinical practice guidelines for chronic kidney disease: kidney disease outcome quality initiative. *American Journal of Kidney Diseases*.

[8] Ergul S, Parish DC, Puett D, Ergul A (1996). Racial differences in plasma endothelin-1 concentrations in individuals with essential hypertension. *Hypertension*.

[9] Laragh JH, Case DB, Atlas A, Sealey JE (1980). Captopril compared with other antirenin system agents in hypertensive patients: its triphasic effect on blood pressure and its use to identify and treat the renin factor. *Hypertension*.

[10] Weber MA, Black H, Bakris G (2009). A selective endothelin-receptor antagonist to reduce blood pressure in patients with treatment-resistant hypertension: a randomised, double-blind, placebo-controlled trial. *The Lancet*.

[11] Krum H, Katz SD (1998). Effect of Endothelin-1 on exercise-induced vasodilation in normal subjects and in patients with heart failure. *American Journal of Cardiology*.

[12] Cardillo C, Campia U, Bryant MB, Panza JA (2002). Increased activity of endogenous endothelin in patients with type II diabetes mellitus. *Circulation*.

[13] Mather KJ, Mirzamohammadi B, Lteif A, Steinberg HO, Baron AD (2002). Endothelin contributes to basal vascular tone and endothelial dysfunction in human obesity and type 2 diabetes. *Diabetes*.

[14] Cardillo C, Campia U, Iantorno M, Panza JA (2004). Enhanced vascular activity of endogenous endothelin-1 in obese hypertensive patients. *Hypertension*.

[15] Lteif A, Vaishnava P, Baron AD, Mather KJ (2007). Endothelin limits insulin action in obese/insulin-resistant humans. *Diabetes*.

[16] Douglas JG, Bakris GL, Epstein M (2003). Management of high blood pressure in African Americans: consensus statement of the hypertension in African Americans Working Group of the International Society on Hypertension in blacks. *Archives of Internal Medicine*.

[17] Elijovich F, Laffer CL (2002). Participation of renal and circulating endothelin in salt-sensitive essential hypertension. *Journal of Human Hypertension*.

[18] Wenzel RR (2005). Renal protection in hypertensive patients: selection of antihypertensive therapy. *Drugs*.

[19] Parving HH, Persson F, Lewis JB, Lewis EJ, Hollenberg NK (2008). Aliskiren combined with losartan in type 2 diabetes and nephropathy. *New England Journal of Medicine*.

[20] Kedzierski RM, Yanagisawa M (2001). Endothelin system: the double-edged sword in health and disease. *Annual Review of Pharmacology and Toxicology*.

[21] Gurley SB, Coffman TM (2007). The renin-angiotensin system and diabetic nephropathy. *Seminars in Nephrology*.

[22] Wilhelm SM, Simonson MS, Robinson AV, Stowe NT, Schulak JA (1999). Endothelin up-regulation and localization following renal ischemia and reperfusion. *Kidney International*.

[23] Hocher B, Thöne-Reineke C, Rohmeiss P (1997). Endothelin-1 transgenic mice develop glomerulosclerosis, interstitial fibrosis, and renal cysts but not hypertension. *Journal of Clinical Investigation*.

[24] Simonson MS, Ismail-Beigi F (2011). Endothelin-1 increases collagen accumulation in renal mesangial cells by stimulating a chemokine and cytokine autocrine signaling loop. *Journal of Biological Chemistry*.

[25] Hocher B, Schwarz A, Reinbacher D (2001). Effects of endothelin receptor antagonists on the progression of diabetic nephropathy. *Nephron*.

[26] Iglarz M, Schiffrin EL (2003). Role of endothelin-1 in hypertension. *Current Hypertension Reports*.

[27] Dhaun N, MacIntyre IM, Kerr D (2011). Selective endothelin-a receptor antagonism reduces proteinuria, blood pressure, and arterial stiffness in chronic proteinuric kidney disease. *Hypertension*.

[28] D’Uscio LV, Moreau P, Shaw S, Takase H, Barton M, Lüscher TF (1997). Effects of chronic ET(A)-receptor blockade in angiotensin II-induced hypertension. *Hypertension*.

[29] D’Uscio LV, Shaw S, Barton M, Lüscher TF (1998). Losartan but not verapamil inhibits angiotensin II-induced tissue endothelin-1 increase role of blood pressure and endothelial function. *Hypertension*.

[30] Emori T, Hirata Y, Ohta K (1991). Cellular mechanism of endothelin-1 release by angiotensin and vasopressin. *Hypertension*.

[31] Herizi A, Jover B, Bouriquet N, Mimran A (1998). Prevention of the cardiovascular and renal effects of angiotensin II by endothelin blockade. *Hypertension*.

[32] Imai T, Hirata Y, Emori T, Yanagisawa M, Masaki T, Marumo F (1992). Induction of endothelin-1 gene by angiotensin and vasopressin in endothelial cells. *Hypertension*.

[33] Rossi GP, Sacchetto A, Cesari M, Pessina AC (1999). Interactions between endothelin-1 and the renin-angiotensin-aldosterone system. *Cardiovascular Research*.

[34] Kawaguchi H, Sawa H, Yasuda H (1990). Endothelin stimulates angiotensin I to angiotensin II conversion in cultured pulmonary artery endothelial cells. *Journal of Molecular and Cellular Cardiology*.

[35] Berk BC, Fujiwara K, Lehoux S (2007). ECM remodeling in hypertensive heart disease. *Journal of Clinical Investigation*.

[36] Bohlender J, Gerbaulet S, Krämer J, Gross M, Kirchengast M, Dietz R (2000). Synergistic effects of AT1 and ET(A) receptor blockade in a transgenic, angiotensin II-dependent, rat model. *Hypertension*.

[37] Gardiner SM, March JE, Kemp PA, Mullins JJ, Bennett T (1995). Haemodynamic effects of losartan and the endothelin antagonist, SB 209670, in conscious, transgenic ((mRen-2)27), hypertensive rats. *British Journal of Pharmacology*.

[38] Ikeda T, Ohta H, Okada M (2000). Antihypertensive effects of a mixed endothelin-A- and -B-Receptor antagonist, J-104132, were augmented in the presence of an AT_1_—receptor antagonist, MK-954. *Journal of Cardiovascular Pharmacology*.

[39] Kohan DE, Pritchett Y, Molitch M (2011). Addition of atrasentan to renin-angiotensin system blockade reduces albuminuria in diabetic nephropathy. *Journal of the American Society of Nephrology*.

[40] Joshi D, Gupta R, Dubey A (2009). TRC4186, a novel AGE-breaker, improves diabetic cardiomyopathy and nephropathy in Ob-ZSF1 model of type 2 diabetes. *Journal of Cardiovascular Pharmacology*.

[41] Cingolani OH, Yang XP, Cavasin MA, Carretero OA (2003). Increased systolic performance with diastolic dysfunction in adult spontaneously hypertensive rats. *Hypertension*.

[42] Pacher P, Liaudet L, Bai P (2003). Potent metalloporphyrin peroxynitrite decomposition catalyst protects against the development of doxorubicin-induced cardiac dysfunction. *Circulation*.

[43] Pacher P, Mabley JG, Liaudet L (2004). Left ventricular pressure-volume relationship in a rat model of advanced aging-associated heart failure. *American Journal of Physiology*.

[44] Westerhof BE, Guelen I, Westerhof N, Karemaker JM, Avolio A (2006). Quantification of wave reflection in the human aorta from pressure alone: a proof of principle. *Hypertension*.

[45] Luna LG (1998). *Histologic Staining Methods of the Armed Forces Institute of Pathology*.

[46] Tofovic SP, Kusaka H, Kost CK, Bastacky S (2000). Renal function and structure in diabetic, hypertensive, obese ZDFxSHHF- hybrid rats. *Renal Failure*.

[47] Grassi G, Schiffrin EL (2010). Media-to-lumen ratio as predictor of renal abnormalities in hypertension: new findings, new questions. *Journal of Hypertension*.

[48] Prabhakar S, Starnes J, Shi S, Lonis B, Tran R (2007). Diabetic nephropathy is associated with oxidative stress and decreased renal nitric oxide production. *Journal of the American Society of Nephrology*.

[49] Schmieder RE, Klingbeil AU, Fleischmann EH, Veelken R, Delles C (2005). Additional antiproteinuric effect of ultrahigh dose candesartan: a double-blind, randomized, prospective study. *Journal of the American Society of Nephrology*.

[50] Peppa-Patrikiou M, Dracopoulou M, Dacou-Voutetakis C (1998). Urinary endothelin in adolescents and young adults with insulin-dependent diabetes mellitus: relation to urinary albumin, blood pressure, and other factors. *Metabolism*.

[51] Viswanathan V (2004). Prevention of diabetic nephropathy: a diabetologist’s perspective. *Indian Journal of Nephrology*.

[52] Qi MY, Liu HR, Dai DZ, Li N, Dai Y (2008). Total triterpene acids, active ingredients from Fructus Corni, attenuate diabetic cardiomyopathy by normalizing ET pathway and expression of FKBP12.6 and SERCA2a in streptozotocin-rats. *Journal of Pharmacy and Pharmacology*.

[53] He H, Liu Q, Shi M (2008). Cardioprotective effects of hydroxysafflor yellow a on diabetic cardiac insufficiency attributed to up-regulation of the expression of intracellular calcium handling proteins of sarcoplasmic reticulum in rats. *Phytotherapy Research*.

[54] Qi MY, Xia HJ, Dai DZ, Dai Y (2006). A novel endothelin receptor antagonist CPU0213 improves diabetic cardiac insufficiency attributed to up-regulation of the expression of FKBP12.6, SERCA2a, and PLB in rats. *Journal of Cardiovascular Pharmacology*.

[55] Amann K, Münter K, Wessels S (2000). Endothelin a receptor blockade prevents capillary/myocyte mismatch in the heart of uremic animals. *Journal of the American Society of Nephrology*.

[56] Gagliardini E, Corna D, Zoja C (2009). Unlike each drug alone, lisinopril if combined with avosentan promotes regression of renal lesions in experimental diabetes. *American Journal of Physiology*.

[57] Macconi D, Ghilardi M, Bonassi ME (2000). Effect of angiotensin-converting enzyme inhibition on glomerular basement membrane permeability and distribution of zonula occludens-1 in MWF rats. *Journal of the American Society of Nephrology*.

[58] Ruggenenti P, Mosconi L, Vendramin G (2000). ACE inhibition improves glomerular size selectivity in patients with idiopathic membranous nephropathy and persistent nephrotic syndrome. *American Journal of Kidney Diseases*.

[59] Sorokin A, Kohan DE (2003). Physiology and pathology of endothelin-1 in renal mesangium. *American Journal of Physiology*.

[60] Amann K, Simonaviciene A, Medwedewa T (2001). Blood pressure-independent additive effects of pharmacologic blockade of the renin-angiotensin and endothelin systems on progression in a low-renin model of renal damage. *Journal of the American Society of Nephrology*.

[61] Saleh MA, Boesen EI, Pollock JS, Savin VJ, Pollock DM (2010). Endothelin-1 increases glomerular permeability and inflammation independent of blood pressure in the rat. *Hypertension*.

[62] Zoja C, Liu XH, Abbate M (1998). Angiotensin II blockade limits tubular protein overreabsorption and the consequent upregulation of endothelin 1 gene in experimental membranous nephropathy. *Experimental Nephrology*.

[63] Remuzzi G, Bertani T (1998). Pathophysiology of progressive nephropathies. *New England Journal of Medicine*.

[64] Lehrke I, Waldherr R, Ritz E (1999). Gene expression of the endothelin system in renal biopsies of proteinuric patients: effect of angiotensin converting enzyme. *Journal of the American Society of Nephrology *.

[65] Peterson RG, Anders AFS, Elazar S (2000). The Zucker diabetic fatty rat. *Animal Models of Diabetes: A Primer*.

[66] Dixon R, Brunskill NJ (1999). Activation of mitogenic pathways by albumin in kidney proximal tubule epithelial cells: implications for the pathophysiology of proteinuric states. *Journal of the American Society of Nephrology*.

[67] Baines RJ, Brunskill NJ (2011). Tubular toxicity of proteinuria. *Nature Reviews Nephrology*.

[68] Yusuf S, Sleight P, Pogue J (2000). Effects of an angiotensin-converting-enzyme inhibitor, ramipril, on cardiovascular events in high-risk patients. *New England Journal of Medicine*.

[69] Hileeto D, Cukiernik M, Mukherjee S (2002). Contributions of endothelin-1 and sodium hydrogen exchanger-1 in the diabetic myocardium. *Diabetes/Metabolism Research and Reviews*.

[70] Witte K, Reitenbach I, Stolpe K, Schilling L, Kirchengast M, Lemmer B (2003). Effects of the endothelin A receptor antagonist darusentan on blood pressure and vascular contractility in type 2 diabetic Goto-Kakizaki rats. *Journal of Cardiovascular Pharmacology*.

[71] Verma S, Arikawa E, McNeill JH (2001). Long-term endothelin receptor blockade improves cardiovascular function in diabetes. *American Journal of Hypertension*.

[72] Dhein S, Hochreuther S, Spring CAD, Bollig K, Hufnagel C, Raschack M (2000). Long-term effects of the endothelin(A) receptor antagonist LU 135252 and the angiotensin-converting enzyme inhibitor trandolapril on diabetic angiopathy and nephropathy in a chronic type I diabetes mellitus rat model. *Journal of Pharmacology and Experimental Therapeutics*.

[73] Neal B, MacMahon S, Chapman N (2000). Blood pressure lowering treatment trialists collaboration. Effects of ACE inhibitors, calcium antagonists, and other blood pressure- lowering drugs: results of prospectively designed overviews of randomized trials. *Lancet *.

